# Unfiltered chronic pain: Insights from women of color through a virtual photovoice study

**DOI:** 10.1186/s12905-026-04486-z

**Published:** 2026-05-01

**Authors:** Rahbel Rahman-Tahir, Jane Prophet, Amna Tanweer Yazdani, Afton L. Hassett, Rachel Gentile

**Affiliations:** 1 Graduate School of Social Service, Fordham University, 113 West 60th Street, New York, NY 10023 USA; 2 Transforming Lives, Sheffield Hallam University, Sheffield, UK; 3 Independent Researcher, New York City, USA; 4 Chronic Pain and Fatigue Research Center, University of Michigan Medical School, Ann Arbor, MI USA

**Keywords:** Community-based participatory research, Chronic pain, Women of color, Pain perception

## Abstract

Photovoice was utilized with two groups of women of color in Ann Arbor, Michigan (*n* = 20) living with chronic pain to foster critical dialog about their experiences. The study aimed to explore and understand their lived experiences through collaborative engagement using a virtual Photovoice. The participants identified key themes, including pain, healing, self-care, overcoming obstacles, nature, and spirituality, which provided valuable insights into their journeys. Participant photographs and dialogues reveal how women of color navigate spaces where their pain is often misunderstood, while simultaneously alleviating pain and reclaiming dignity. To support holistic chronic pain management, systemic reforms must cover evidence-based complementary therapies, expand behavioral health access, fund community support groups, and mandate provider bias training co-designed by patients. These efforts should be guided by trauma-informed and interdisciplinary care models, supported by public awareness campaigns that validate the multidimensional reality of chronic pain and address structural barriers to wellness.

**Supplementary Information:**

The online version contains supplementary material available at 10.1186/s12905-026-04486-z.

## Background

 Chronic pain, defined as pain lasting more than three months, affects 20.9% of U.S. adults, with 6.9–7.8% experiencing high-impact pain that significantly limits daily activities [1, 2]. Women are disproportionately affected, experiencing higher rates (20.5% vs. 18.8%) and greater pain intensity than men [2, 3]. Gendered norms often pressure women to normalize their pain, while stigma discourages open discussions [4, 5]. Women of color face additional burdens: for example Black women report greater functional impairment and emotional distress [6]. Intersecting social, cultural, and familial expectations frequently compel women to manage pain in silence [4, 5].

Disparities in pain assessment and treatment disproportionately affect minoritized racial and ethnic groups [7, 8]. For Black, indigenous, and people of color (BIPOC) individuals, chronic pain is shaped by interconnected biological, psychological, and structural forces - ranging from social determinants of health and epigenetic stressors to entrenched racial discrimination [9]. Racial discrimination exacerbates pain experiences: 50–75% of Black, Latin, and Asian individuals report discrimination in clinical settings [8], contributing to 20% healthcare avoidance among Black patients [8, 10]. Many women of color encounter clinicians who doubt them and dismiss symptoms as psychological, or are inaccurately viewed as drug-seeking [11–13]. Historically, women have also been pathologized through diagnoses such as ‘hysteria’, a label rooted in gendered bias [14]. The “strong Black woman” stereotype, further minimizes Black women’s pain and discourages disclosure [15]. Misconceptions persist among clinicians, 40% of whom endorse false beliefs about racial differences in pain perception, contributing to delayed diagnoses and inadequate treatment for Black patients [16]. Consequently, Black patients are less likely to be prescribed pain medications and, when they are, often receive lower doses.

Healthcare providers frequently dismiss or minimize the pain of socioeconomically marginalized women, and BIPOC patients pain is routinely underassessed, leading to poorer outcomes [8, 17]. Provider bias, language barriers, and systemic inequities intensify these disparities: only 20% of pain specialists speak Spanish, limiting care for Spanish-speaking patients [18], and reliance on Medicaid or Medicare limits access to specialized treatments for many Black and Latina patients [7]. These systemic barriers delay pain treatments, underscoring the need for systemic change to address the intertwined physical and social drivers of inequity.

Clinical approaches often overlook the daily impacts of chronic pain, particularly for minoritized groups. Participatory Action Research (PAR) centers patients voice and lived experiences, revealing effective nonclinical interventions missing from conventional care [19, 20] and highlighting barriers to wellness such as stigma, limited healthcare access, and culturally insensitive practices. In doing so, PAR can enhance more equitable and holistic care for diverse populations [21]. Photovoice, a PAR method developed by Caroline Wang and Mary Ann Burris (1997), uses visual storytelling to document experiences, highlighting individual and shared challenges while building supportive communities and fostering empathy among participants, providers, and researchers [22, 23]. Photovoice repositions participants as co-researchers by inviting them to document and reflect on their experiences through photography [22]. Photovoice integrates three theoretical foundations: feminist theory in highlighting how social and political structures reinforce gendered inequalities [24]; Freirean pedagogy in using participant-generated images to spark dialogue, build critical consciousness, and support action; and documentary photography in treating participants as experts of their own lives [25]. Therefore, Photovoice advances the democratization of knowledge as a form of social justice [26]. It does so by enabling community members to create photo/text narratives that showcase their strengths and concerns, raising public awareness and inspiring action [22, 23]. The process involves three key steps: 1) empowering participants to document and reflect on community strengths and challenges, 2) fostering critical dialogue through group discussions of images, and 3) presenting insights to policymakers to advocate for change [27].

Initially, Photovoice was used with women in rural Yunnan, China, to help them communicate and advocate for their reproductive health needs [23]. Globally, Photovoice has been used to understand public health challenges ranging from infectious diseases like COVID-19 [28], HIV [29], and Tuberculosis [30] to complex psychosocial issues such as mental health disorders [31] and substance use [32]. Photovoice is widely used in work with marginalized communities because it offers a low-burden method that can foster participant empowerment [33], as shown in research with women across diverse marginalized contexts, including those living with HIV/AIDS [34], disabilities [35], discrimination faced by Aboriginal women [36], homelessness [37], and pregnant or postpartum challenges [38].

Photovoice provides a nuanced understanding of lived experience that traditional quantitative and qualitative methods often miss, offering researchers and clinicians insights into pain that might otherwise remain hidden [39]. While Photovoice has been used to explore how patients self-manage invisible illness [40], studies have not focused on patients with intersectional identities. Our study addresses this gap by enabling women of color living with chronic pain to reflect critically, share their experiences, and articulate the intersecting challenges of their daily lives. During the COVID-19 pandemic, Photovoice was adapted to virtual platforms, overcoming barriers such as mobility limitations and geographic isolation [9]. This study employed a virtual Photovoice approach to explore the experiences of 20 women in Ann Arbor, Michigan, living with chronic pain, using a model that combined synchronous Zoom sessions with asynchronous Canvas activities.

## Methods

### Setting

Two researchers and a coordinator codesigned the study in collaboration with an advisory board of patients, caregivers, community members, and pain experts to understand the lived experiences of women with chronic pain. The study’s format that combined synchronous sessions on Zoom with asynchronous activities on Canvas, accommodated COVID-19 constraints.

### Sampling and recruitment

The advisory board recruited participants through print advertisements, social media, and online forums. The inclusion criteria for the study included Michigan residents who self-identified as women of color, and those who experienced chronic pain for three or more months, assessed through self-reported diagnoses and medical histories. Of 21 women recruits, one withdrew due to time constraints, leaving 20 participants. Two focus groups, consisting of 8 to 10 participants each, were chosen for this study because this multi-focus group approach is increasingly being employed in health care research to investigate under-studied topics. Participants were assigned randomly to two groups that met on Monday and Tuesday evenings. Availability sampling had been agreed in principle by the research team, one participant could only attend on a Tuesday evening, so that participant was not randomly assigned.

Onboarding included completing consent forms and procedural discussions. Seven participants used study-provided digital cameras and thirteen used smartphones for photography. The digital cameras were KODAK DA00236 - KODAK M38-35 mm Reusable Camera, Lens, Built-in Flash, AA Battery). The smartphone specifications included simply needing manual controls to turn off automatic processing.

A virtual orientation explained: the format of planned synchronous Zoom sessions with asynchronous Canvas activities, compensation ($50 per session), and overall Photovoice process [22]. Before the first session, participants completed a sociodemographic survey, watched introductory videos, and reflected on their initial impressions of Photovoice. Specifically, the videos introduced the research team, explained the principles of Photovoice, addressed ethical considerations, and offered practical photography tips.

### Photovoice group procedures

**Fig. 1:**
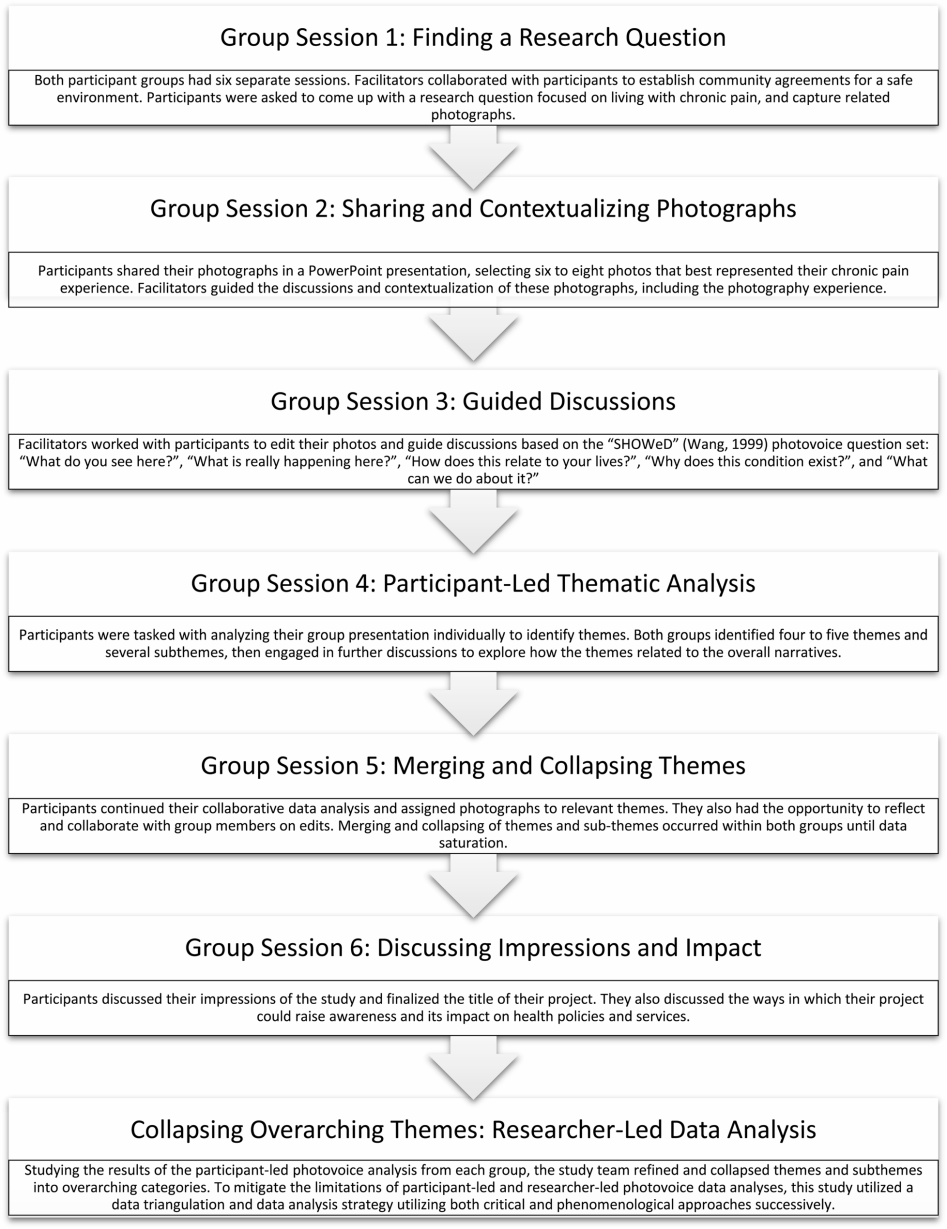
Photovoice study procedures and process

Participants took photographs and wrote corresponding narratives in response to their research questions, then discussed them in focus groups. Participants collaboratively analyzed that data, identified themes across images and narratives and organized data accordingly. The process unfolded in six sessions comprising: Photovoice training, developing research questions, photography, collaborative analysis, and final reflection [41]. Groups A and B met separately on Zoom for six sessions, facilitated by the researchers and coordinator. Figure 1 summarizes the procedure.

*Session One* included asynchronous video training led by an art professor on photographic interpretation, including the emotional effects of camera angles and lighting [42]. Critical dialogue began in the first synchronous Zoom session through participatory practices that redistributed power and centered participants lived experiences, grounding the study in a critical framework from the outset. Together, participants developed community guidelines–that emphasized active listening, respect for individual truths, and equal sharing, shifting authority from researchers to participants and establishing conditions for safe, respectful, and equitable critical exchange [39]. The session continued with an icebreaker when participants shared experiences of chronic pain and initial impressions of Photovoice to ground the process in their lived expertise, ensuring they shaped the subsequent research. From this dialogic foundation, each group collaboratively developed a research question central to living with chronic pain. Group A’s question was: “What are daily challenges and adaptive strategies of living with chronic pain that providers often fail to see or understand?”. Group B asked, “How do race, gender, and other identities shape unseen challenges and daily strategies in chronic pain management?” Two weeks of individual photography followed in response to the group’s research question. Group A produced 15–50 images and Group B created 6-100. Participants uploaded curated selections (6–20 photographs and 6–50 respectively) to a shared platform.

In *Session Two*, participants collaborated to present their images in group PowerPoint. Facilitators used the ORID (Objective, Reflective, Interpretive, Decisional) framework [40] to guide participants discussion to categorize photographs, reflect on challenges, such as time and health constraints, and describe emotions involved in the process. Each participant selected 6–8 key images that best represented their chronic pain experience, prioritizing one photograph. They then completed a reflective video assignment on the meanings of their chosen images, taking additional photographs as needed.

In *Session Three*, facilitators helped participants to edit their selected photographs, cropping, collaging, and adjusting lighting and/or color as participants directed. They then guided discussions using Photovoice’s “SHOWeD [43] framework (“What do you see here?”, “What is really happening here?”, “How does this relate to your lives?”, “Why does this condition exist?”, and “What can we do about it?”).

In *Session Four*, participants individually analyzed group presentations, identifying themes for group discussion. Groups A and B identified five and four themes respectively, along with sub-themes (Table 1) and explored how they related to these overall narratives.

**Table 1:** Themes and Sub-themes for Groups A and B

Group A	Group B
*Spectrum of Healing*	*Breakthroughs/Overcoming Pain*
Self-Care	Family, Nature, Outdoors
Complementary Medicine	Moments of Pain and Relief
Pre-Planning	Obstacles and Everyday Activities that can be Difficult
Improving Environment to Feel Better	Hindrances
Distractions	Movement
Faith	Mobility
Symbols of Anti-Western Medicine	Motivation
*My Truth* vs. *Perception*	Helps
My Truth	*Strength/Perseverance*
Assumptions	Medication
Stereotypes	Activities
Body Shaming	Positive Coping
*How we feel our pain/Lived Experience of pain/Embodiment of pain*	*Spirituality*
Isolation vs. Looking Out	Positive Impact of Nature, Family, Social/Emotional Support
Patient-Provider Engagement	Hope
*Distractions*,* Contradictions*,* and Juxtapositions Overcoming Barriers*	Things that Bring Joy
Not being able to do things we used to do	Power of Faith
	Peacefulness and Relaxation
	Juxtaposition of Hope with Pain
	*Pain*
	Grounding
	Healing
	Breakthroughs
	Adaptability

In *Session Five*, both groups continued data analysis to construct a cohesive narrative, collaboratively assigning photographs to themes and making revisions. This participant-led process fostered ownership of the research analysis. Themes and subthemes were merged or collapsed until no new themes emerged; facilitators determined that data saturation had been achieved [43]. The collaborative analysis process adopted an inductive, participant-led thematic approach.

Each group’s thematic structures (Table 1) informed iterative analysis. Group A developed five themes with 13 subthemes (e.g., “Spectrum of Healing,” “How We Feel/Embody Our Pain”), while Group B developed four themes (e.g., “Strength/Perseverance,” “Spirituality”) with 21 subthemes. The study team synthesized and refined these findings into overarching categories, comparing patterns across groups, then returned that to participants for feedback, maintaining CBPR’s emphasis on participant empowerment alongside scholarly rigor. Final collaborative synthesis consolidated themes and subthemes detailed in the following “Results” section.

In *Session Six*, participants reflected on the Photovoice process, selected project titles, and discussed future directions. Citing COVID-19 health risks, participants chose to share their work via a hardcover and eBook rather than through public exhibition, aiming to raise awareness among healthcare providers, policymakers, and others about biases in pain treatment. Group A titled their book “Journeying Beyond Pain: Our Truth as Women of Color” (Group A) and Group B “The Invisible Struggle”. Participants organized photographs and narratives according to their original themes and subthemes (Table 1) while the three overarching categories were developed for academic dissemination. After their final session, each participant received an evaluation survey which included a 5-point Likert scale and two open-ended questions; fourteen completed it. The survey measured participant perceptions of facilitator effectiveness, confidence in communicating about chronic pain, comfort with the online group format, and the overall perceived value and potential health impact of the program, while also gathering qualitative feedback on strengths and areas for improvement. Six participants did not respond to reminder emails to complete the form. Of those, three participants accessed the form but did not complete it. No reasons for non-completion were provided.

## Results

Groups A and B each included five African American/Black women and one Asian woman, with three Latin American and five Middle Eastern/South Asian participants across both groups. Participants ages ranged from 20 to 68 years, and their experiences of chronic pain varied, including migraines, back pain, fibromyalgia, arthritis, and multiple sclerosis. Including women of color with such a range of chronic pain experiences allows for findings to be generalizable to the diverse realities of this population that have been historically underrepresented in research [44].

Across both groups, participants characterized their lived experience of chronic pain using three overarching themes: 1) its mental and physical toll, 2) modalities for healing and alleviation, and 3) the impact of others perceptions and attitudes.

### The mental and physical toll of living with chronic pain

**Fig. 2:**
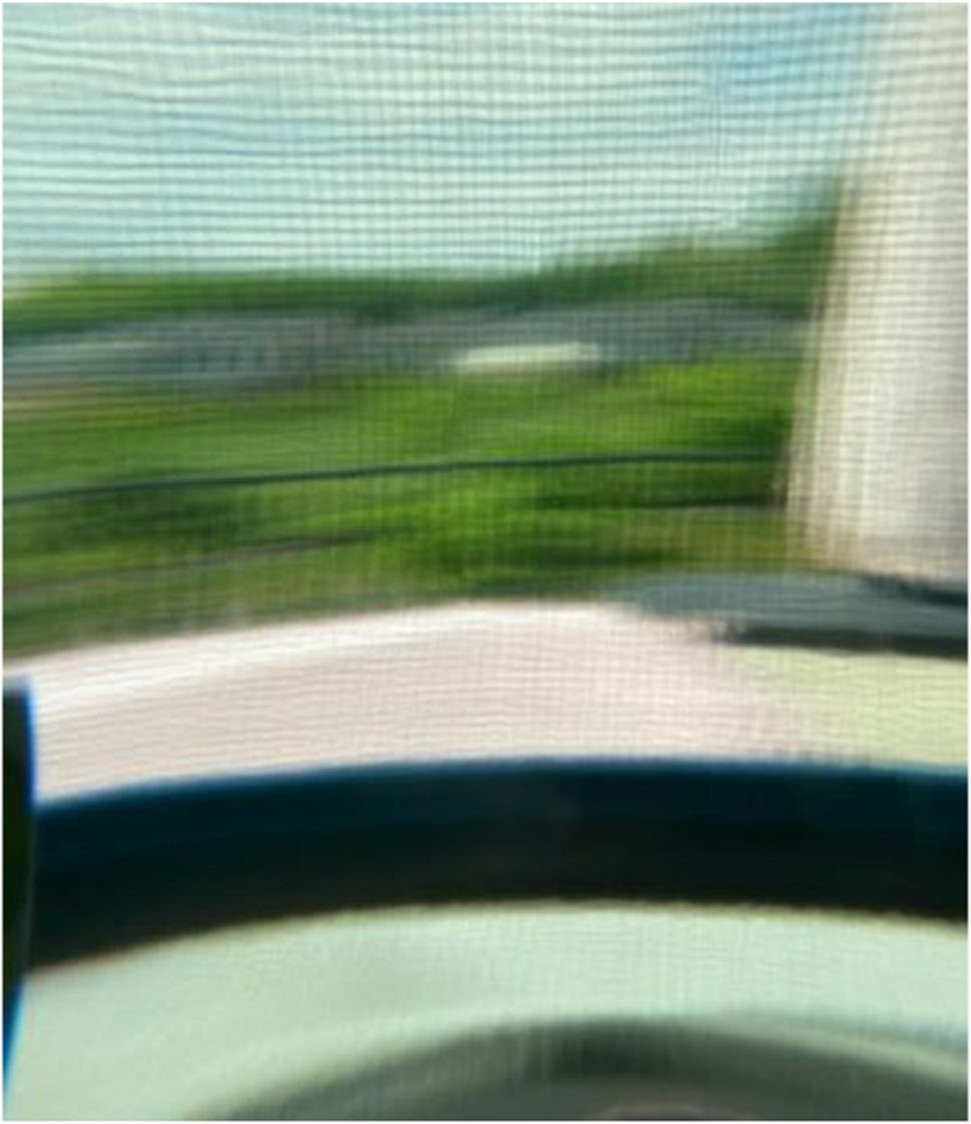
One of the pains that I have are like chronic migraines and I tried to take like these photos basically like what my vision looks like as I’m going through these migraines. This image is like very blurry on purpose

**Fig. 3:**
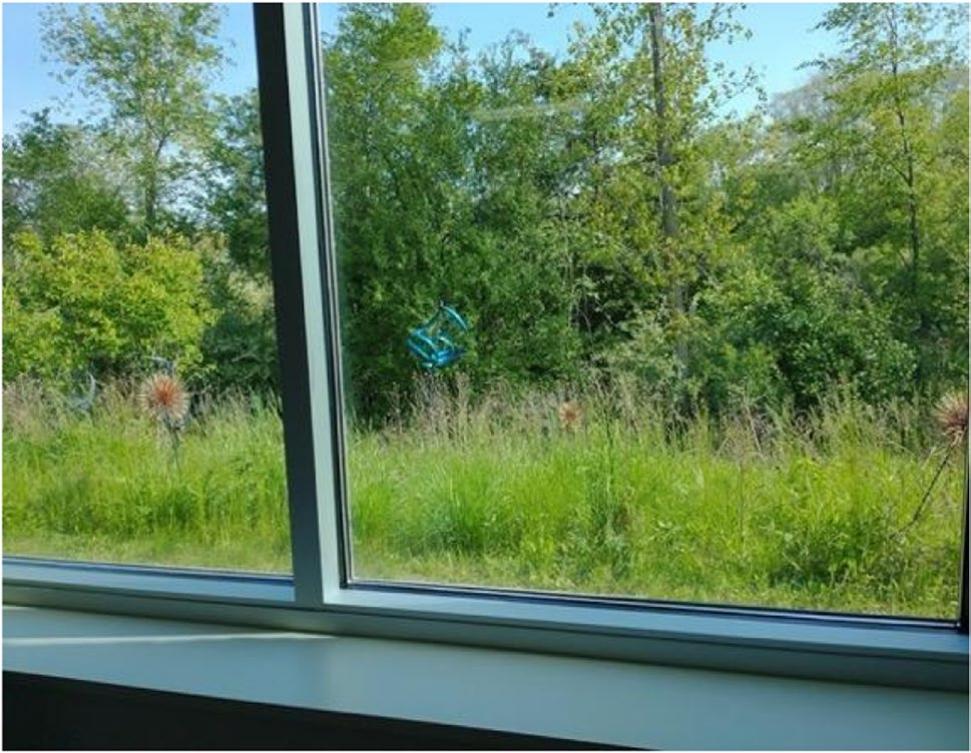
Looking from the inside, out. In many cases, pain causes me to be sidelined more than I would like to be. So I find myself looking out at things, instead of being a part of them. I’m not an active participant in what’s going on, and it makes me sad and lonely sometimes

Photographs and discussions across participant groups highlighted the mental and physical impacts of chronic pain. Physical symptoms, restricted mobility, and accessibility challenges shaped daily life, compounded by mental health struggles and social isolation. Photographs depicted concrete and abstract expressions of pain, for example, a blurred window view illustrating vision during a migraine (Figs. 2 and 3).

In subtheme “*Isolation / Us looking out*,” participants expressed being sidelined from activities they once enjoyed, resulting in diminished quality of life. Photographs included unused tennis balls from a participant no longer able to play and views of and from bedroom windows symbolizing confinement while family enjoyed being outdoors. Mental health challenges (depression, low self-esteem, isolation and shame) frequently surfaced in participants narratives:

*When people stare*,* it makes me feel ashamed. However*,* I should not be ashamed as this is NORMAL for people with chronic conditions.*

**Fig. 4:**
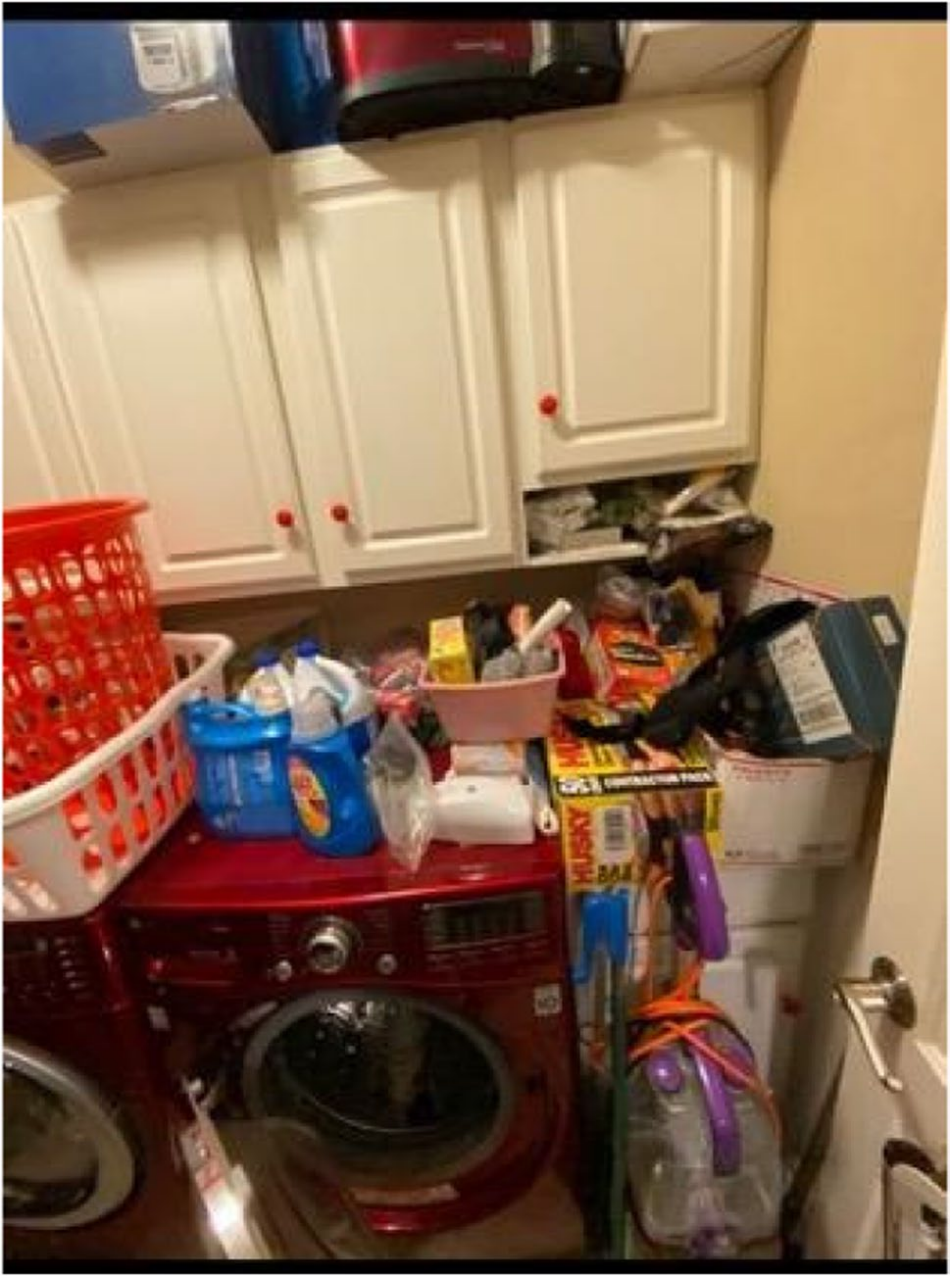
This is a reminder of what I need to do. Also lets me know that if it’s not clean, it’s because of the amount of pain I’m in

Participants reported numerous hindrances and struggles in living with chronic pain, including frustration over lost independence and reliance on others. One shared a photograph of her laundry room, noting that household tasks accumulate when pain limits her (Fig. 4).

Other photo/texts examined accessibility barriers, such as limited affordable public transport, that compound the financial burden on those with accessibility needs. Participants also described exclusion from public spaces where pain triggers, like bright lights, are unavoidable; one photographed her hand blocking a light fixture, explaining:

*When you’re in your own space*,* you can control and prevent triggers*,* but when you go out into the world*,* it is not always accommodating to your conditions in public spaces; it is hard to accommodate everybody. But I think there needs to be work done to make areas more accessible or comfortable for people with disabilities or people with certain conditions.*

### Modalities for healing and pain alleviation

Participants discussed the physical toll of chronic pain and the measures they are compelled to adopt when conventional medicine falls short. Healing was described as multifaceted and individualized, often involving complementary therapies and traditional remedy alternatives to Western medicine. One participant photographed her “pain-fighting kit,” a collection of resources she relies on to manage her pain.

*This roller is a new addition to my pain fighting kit. I like to roll this under my feet*,* and it relieves some of the tightness and pain under my feet. This is not a miracle worker*,* but it is definitely a valuable tool in my fight against pain.*

**Fig. 5:**
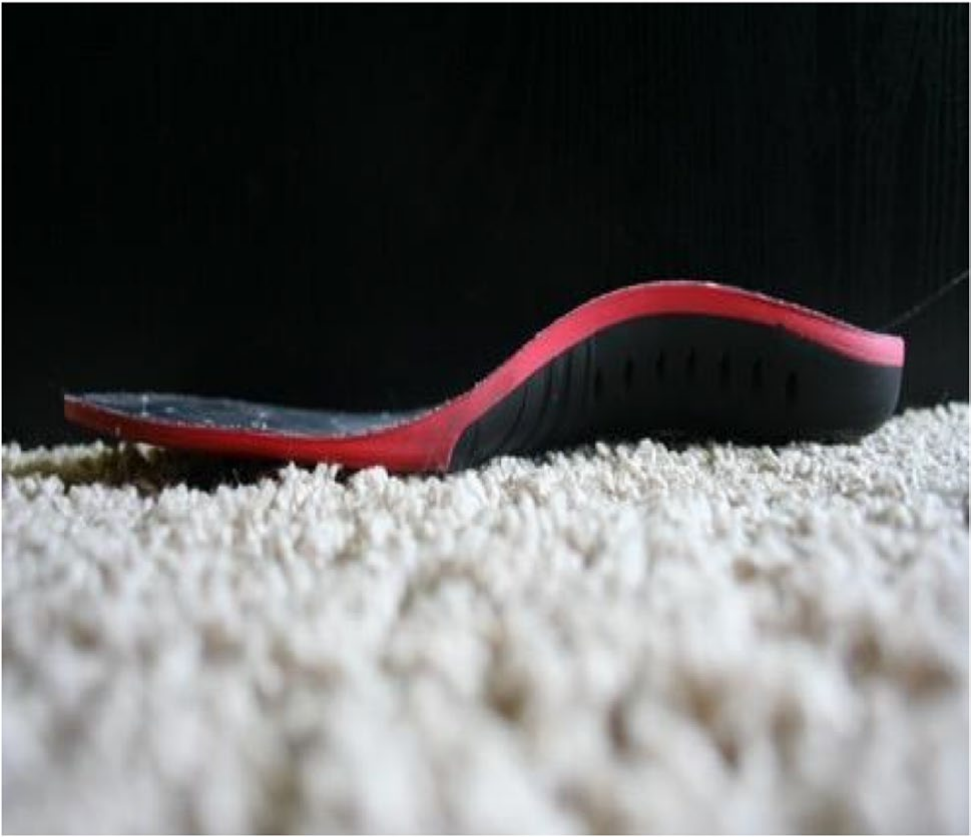
These are my insoles…I always joke with my mom that I inherited her feet and her joints because we both have flat feet and joint pain

Several image‒text combinations highlighted participants pain management strategies. One shared a self-portrait during a painful iron infusion, explaining that mindfulness and meditation it. Another photographed insole (Fig. 5) that alleviate joint pain, allowing her to wear preferred shoes. A third emphasized the importance of proactive preparation for flare-ups:

*I have a lot of like casts and bandages at ready*,* because sometimes*,* especially on days where I’m super active*,* it can flare up. Especially with my ankles and my wrists*,* so those are casts that I have on standby whenever I need … truly*,* that situation where it is something that you just have to live with*,* and you just cannot cancel last minute things always.*

**Fig. 6:**
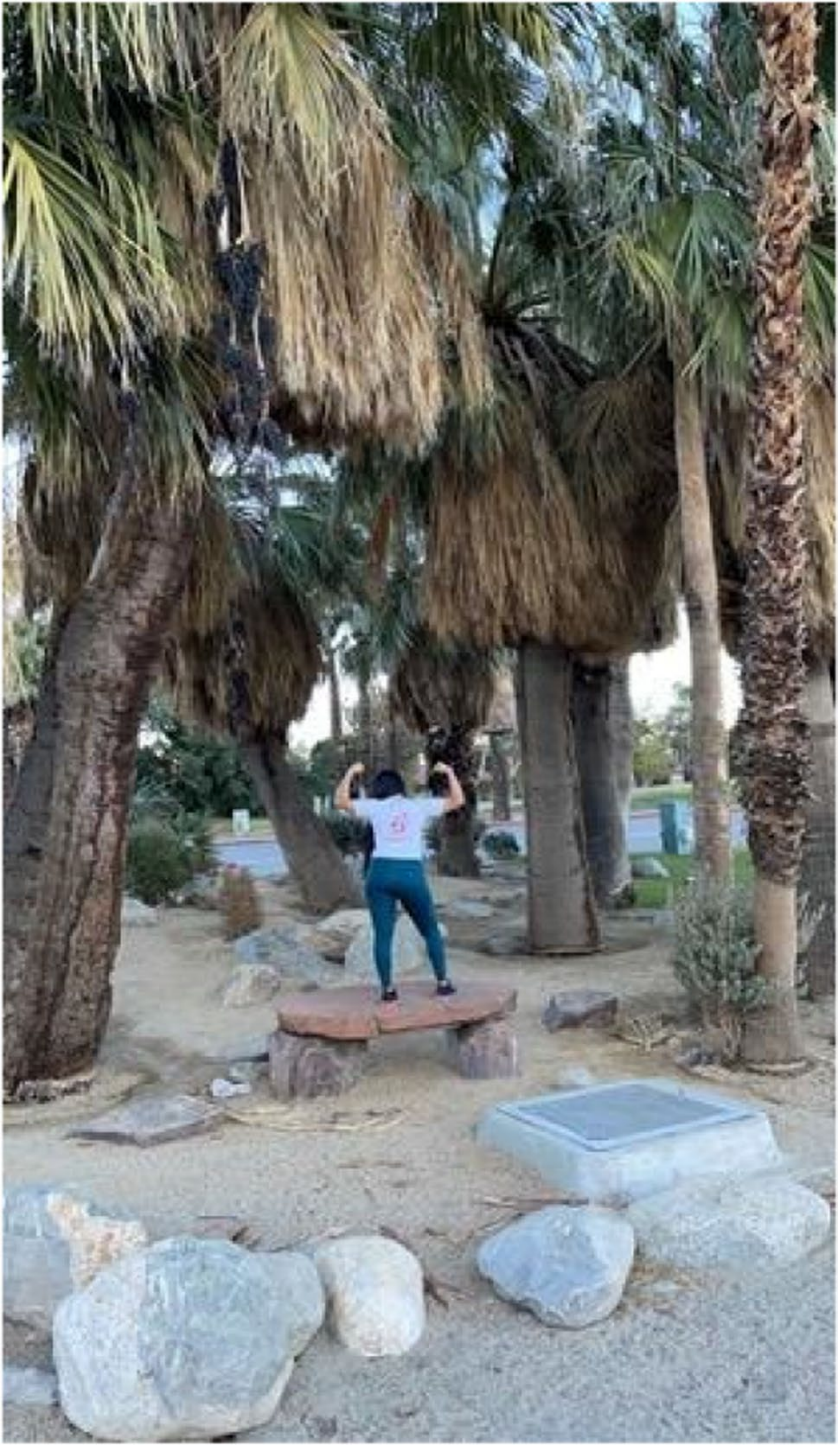
Physical activity helps alleviate the pain that I experience with my condition. When I work out or do a form of physical exercise, I escape my chronic condition mentally. I am also able to push my body physically, which makes me feel like I have no chronic condition at all. On this day, I was feeling strong, both mentally and physically

**Fig. 7:**
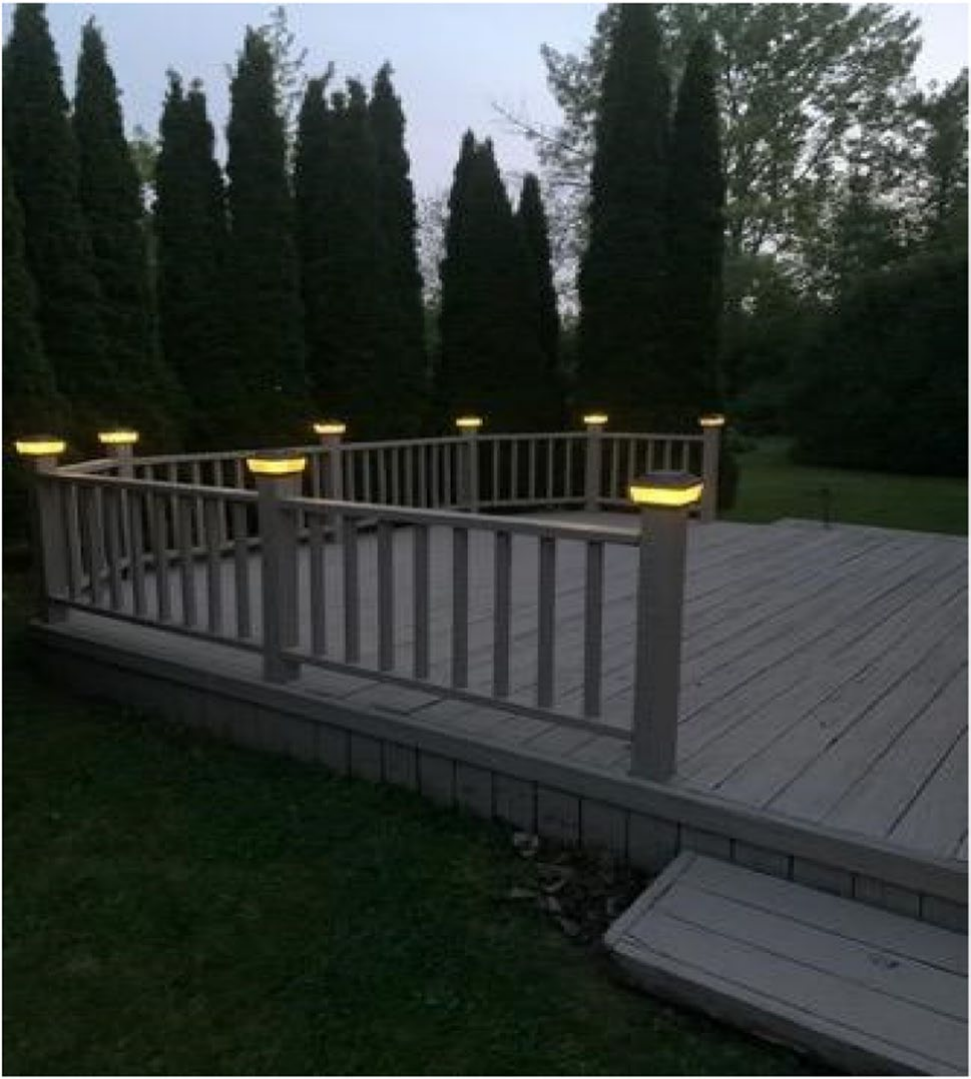
I always mowed my own lawn and created the landscape that I wanted to see. I created my own world to make myself happy. I cannot do that planting now, but it always makes me happy to look at it

Participants identified movement and physical activity, such as hiking, walking, yoga, meditation, biking, and general exercise as important self-care strategies. One illustrated the connection between exertion and wellness (Fig. 6), while others emphasized the healing power of enhancing one’s environment to improve mood (Fig. 7).

**Fig. 8:**
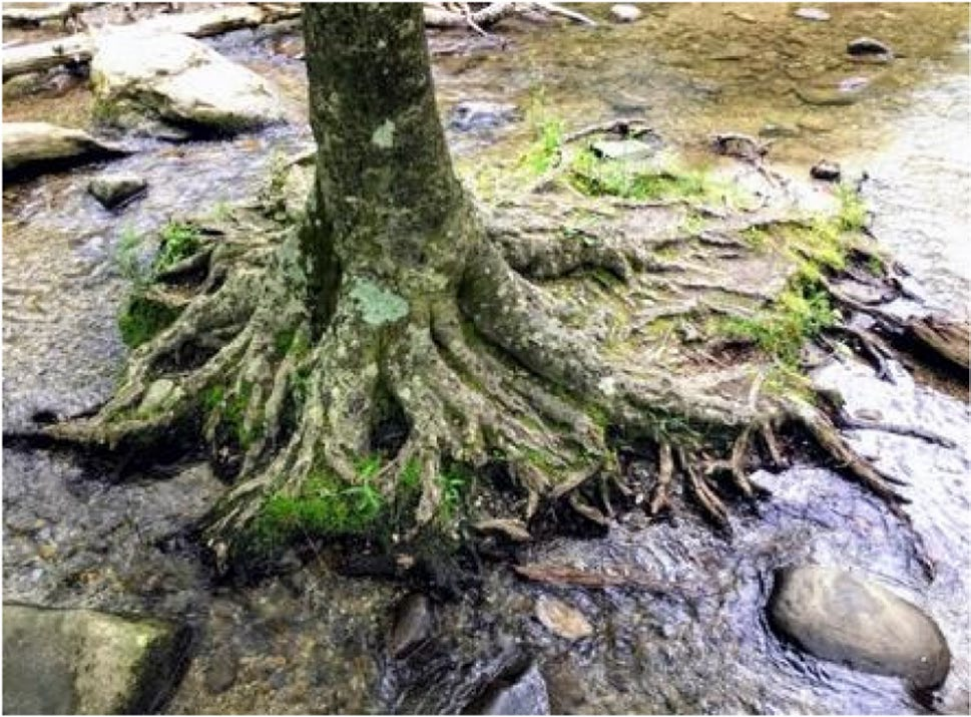
I get a lot of nerve pain, both from nerve damage in my leg and then just from like pinched nerves in my lower back from the way that I walk unevenly and do things unevenly. Being there, it was truly nice to watch that cold water just rush over the roots but have the tree still be standing, even though it has all these exposed roots and it made me think about nerves - like the feeling that comes over it - it’s like when I get nerve pain … I kind of stop doing everything that I do right there and that is kind of what I think about when I imagine cold water rushing over you know something like this, something that is alive and thriving in it

Nature’s positive impact was a recurring theme: some mourned their inability to engage in outdoor activities, whereas others found peace and healing in nature’s beauty. One participant connected tree roots to her nerve, noting that their resilience mirrored her capacity to endure and thrive despite pain (Fig. 8).

Another participant juxtaposed pain and nature:

*I really liked the moment*,* right after the rain. Just that pop of green and the smell*,* you know*,* of earth. You’re walking or just doing things*,* sometimes you forget about the pain. And sometimes you forget about those beautiful plants and then you see them*,* so I feel it is kind of the same thing; these are beautiful things that we should stop and look at. Just like pain*,* I guess*,* we must accept it too.*

**Fig. 9:**
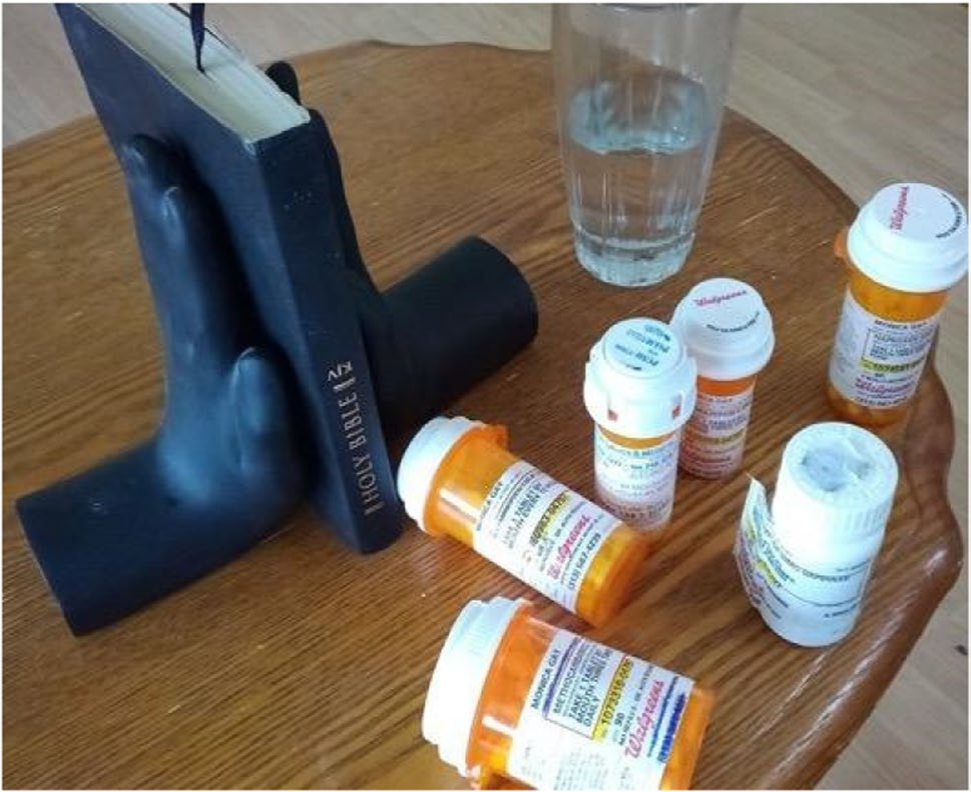
I have a brain injury which has caused my eyes and brain to no longer work together (creating misaligned eyes, double and blurry vision). So emotionally that’s extremely painful, There’s no medicine for that. […] And so, medication alone cannot do it, spirituality and religion in my community, these are factors in terms of how many of women African Descent deal with pain

Participants described faith and spirituality as key to bolstering inner strength and hope as they healed, exemplified by a bible photographed next to medications (Fig. 9).

### Impact of the perceptions and attitudes of others on the lived experience of chronic pain

Participants discussed how misconceptions, stereotypes, and biases from health care providers, family, and the public impacted their journey. They highlighted disconnects between their internal reality and how they are perceived, as discussed in three ways: invisibility of their pain, gendered and racial stereotypes that dismissed their suffering, and medical bias that wrongly attributed symptoms or dismissed them entirely. One participant expressed the exhaustion of maintaining ‘normal’ appearances, stating:

*Please know that you cannot look at someone and say “you look okay*,*” you cannot do that. You cannot see inside of my body*,* my mental anguish*,* emotional suffering*,* but I can feel everything - leaving me in grief over a full and vibrant quality of life I will never have again. So if you see me smile*,* I’m just trying to look normal*,* just for one moment.*

Participants described the pressure to remain productive and counter racial and gender stereotypes of being “lazy”. Another participant expressed a shared sentiment among women of color: the lack safe spaces to be vulnerable, which was then backed up by other participants.

**Fig. 10:**
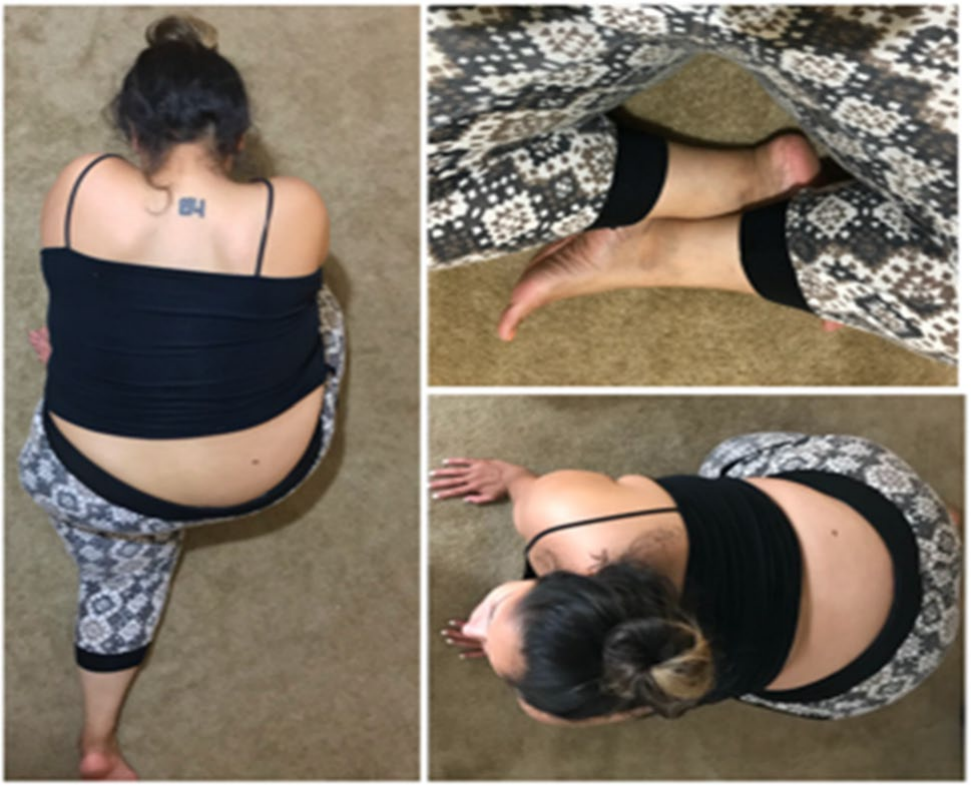
My fat body. I did not want to care of it. I punished it with lack of love and attention…My body was not loved and maybe that is where the pain comes from: lovelessness. Maybe if I was loved—maybe if I loved it more then maybe it would hurt less

**Fig. 11:**
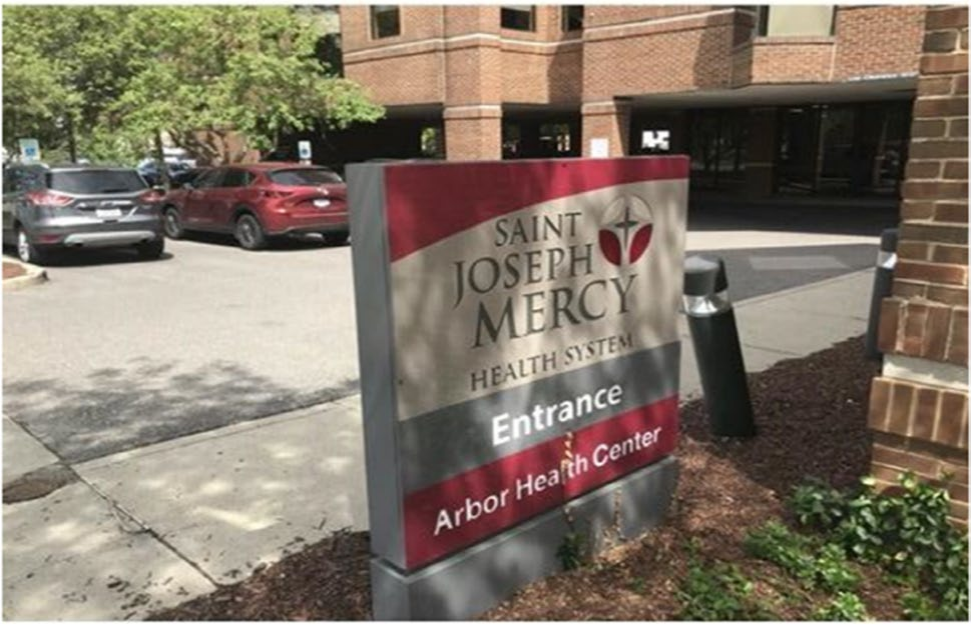
This is the place where ‘the magic happens.’ I am fortunate to have good healthcare insurance, great providers, and minimal difficulty reaching my providers or their staff. There have been appointments where I did not receive good news but I left the visit confident that my care was in very capable hands. It truly matters when a provider has a good bedside manner, is familiar with your illness, and is considerate. It can ease a patient’s concerns and that’s a large part of the battle – easing and comforting the patient…helping the patient to feel safe

The critical role of patient-provider interactions could be further invalidating or, conversely, provide validation and safety. Invalidating experiences included bias, described by one participant as “everything is attributed to my fat body [by the clinician]” (Fig. 10). In contrast, a positive care experience was highlighted as foundational: “It truly matters when a provider has a good bedside manner… helping the patient to feel safe” (Fig. 11).

## Discussion

Our findings underscore that chronic pain is a biopsychological, not exclusively biomedical. Participants photographs, texts, and discussions reveal how women of color navigate spaces where their pain is often misunderstood, while working to alleviate pain and reclaim dignity. These results highlight the need for patient-centered care and for empathetic social networks that acknowledge social determinants of pain and validate lived experience.

Because chronic pain is often ‘invisible,’ it is frequently dismissed, intensifying the challenges faced by those who live with it [45, 46]. Participants used visceral metaphors blurred images representing migraines to render this invisibility visible. Their physical suffering was compounded by depression, shame, and isolation, reflected in images of being confined indoors. Many described a disconnect between their internal reality and how they are perceived, often internalizing stereotypes that cast them as exaggerating, “lazy” or not credible. Research shows that invalidating pain increases pain severity, loneliness, and depression [39]. This invalidation functions as a form of gendered, disenfranchising talk that discredits women’s concerns pressures them into silence. This leads to internalized shame, guilt and self-doubt [47]. These challenges intensify when pain limits social engagement, increasing the risk of depression [48]. Guilt over perceived inadequacy or unmet expectations correlates with greater pain intensity and reduced functioning [48].

Echoing prior research [49], participants described gendered and racialized pressures to appear strong and minimize pain, resulting in withholding disclosure to clinicians, family, and friends. Participants health-seeking behaviors and internalized pain narratives reflected culturally-prescribed gender roles and expectations of stoicism [50]. The burden of chronic pain is further intensified by the gendered organization of care, in which women often subordinate their own needs to the demands of family life [37, 51]. Participants described powering through pain to fulfill caregiving and household responsibilities, downplaying their suffering for others comfort. Patterns of invalidation extended to clinical encounters, exemplified by a participant whose provider dismissed her symptoms by attributing them simply to her weight.

In response to dismissal by clinicians, family, and colleagues, participants demonstrated agency through adaptive pain-management strategies. Healing and daily survival emerged as active, holistic negotiations with their pain. Self-management extended beyond physical tools and therapies to encompass movement, nature, spiritual faith, creative acts, and social support; aligning with evidence that women often turn to faith, rest, community, and alternative treatments as vital sources of relief and control [32, 52].

Participants reported that the focus groups helped shift chronic pain from a private struggle into a shared, agential process. Photovoice functioned as a research tool and a reflective practice, empowering participants to depict the subjective nature of their pain, foster community, and raise awareness through sharing e-books with health care providers, family and friends. In the summative evaluation 77% (*n* = 16) agreed the project provided knowledge to improve their health, and 69% (*n* = 14) learned new ways to communicate their pain. The study recommends investment in structured support groups and patient-advocacy resources, which offer validation and practical tools for navigating healthcare systems [53]. This is an important finding given that perceived social support mitigates depressive symptoms linked to pain invalidation [54].

While the study identified the layered realities of women of color navigating chronic pain, there are number of limitations to the study. The sample consisted of 20 women of color in Ann Arbor Michigan, which restricts generalizability to other geographic contexts such as rural or suburban populations. Also, relying on Zoom for data collection presumed participants had reliable technology and internet bandwidth, effectively excluding those facing further structural determinants. Self-selection bias is also a limitation as voluntary participation to the study may have drawn women with greater agency. The recruitment strategy was developed by a project advisory board that included nine compensated patients, caregivers, community members, and faculty from the University of Michigan’s human services division. Future research could mitigate self-selection bias by partnering with coalitions and community organizations to actively recruit participants facing greater isolation or structural barriers. To address these sampling and access limitations, future research should prioritize hybrid recruitment strategies across varied geographic settings and intentionally invest in technology access (beyond offering cameras) to ensure that those most marginalized by structural barriers are not inadvertently excluded. Longitudinal studies across different geographic contexts are likewise needed to evaluate culturally rooted therapies and measure whether sustained pain validation over time meaningfully affects mental wellbeing and quality of life. Finally, while this study demonstrated the potential of Photovoice and storytelling among women of color experiencing chronic pain, future research should assess its viability as clinical interventions that can be systematically integrated into care settings. Such work would examine how these approaches bridge patients “invisible” lived experiences with clinical practice and help mitigate the harms of systemic disconnect.

To sustain the holistic strategies identified by participants, insurance and public health systems should cover evidence-based complementary therapies and expand behavioral health supports. Community-based programs that cultivate social connection are likewise essential to counter isolation. Medical providers should engage in mandatory, co-designed implicit bias trainings to dismantle gendered and racial prejudices in pain assessment, and center patient narratives as primary evidence. Such trauma-informed systemic changes are crucial to preventing traumatization and medical mistrust among women of color, whose pain is often interwoven with histories of trauma, violence, and discrimination [48, 49].

Managing the biopsychosocial realities of chronic pain requires an interdisciplinary team (physicians, psychologists and social workers) integrating biomedical care, addressing mental health and internalized stigma, and helping patients navigate structural barriers like childcare. Physical and occupational therapists, and complementary or integrative medicine providers can support this core team. Awareness campaigns are needed, informed by patient-centered methods like Photovoice, to educate the public, employers, and informal support networks about the legitimacy of invisible illnesses, the harms of invalidation, and the multidimensional nature of chronic pain.

Because the study found that women often put their family’s needs ahead of their own pain, any intervention should use participatory approaches that help women see taking care of themselves as a necessity, not something selfish. Facilitated peer groups would give women space to name the pattern of “powering through” pain and learn new ways of coping, while offering childcare and flexible schedules removes practical barriers to attending. These spaces could connect with others with similar experiences and incorporate the strategies that the women used in the study like movement, time outside, faith, creative activities. By turning the burden of care from something women carry alone into a shared experience with support, this approach reflects the same sense of connection and healing that came out of the Photovoice project.

## Conclusion

Using Photovoice, this study captured many complex physical and emotional experiences of women of color living with chronic pain, fostering connection and therapeutic benefits through shared narratives. It amplified marginalized voices to highlight systemic disparities in pain care and underscored the need for trauma-informed practices, policy reforms, and greater accessibility to complementary medicine resources. The findings call for systemic change in chronic pain management, centering patient experience promoting equity and strengthening community empowerment. Methods such as Photovoice offer a pathway toward a more inclusive and responsive approach to chronic pain care.

## Supplementary Information

Supplementary Material 1.

## Data Availability

All relevant data, transcripts, and study tools used to support the findings of this research can be provided upon reasonable request to the corresponding author. The data is not publicly available currently due to ongoing analysis and the preparation of additional manuscripts.

## Abbreviation

BIPOC: Black, indigenous, and people of color individuals
