# Supplementary material for: Unfiltered chronic pain: Insights from women of color through a virtual photovoice study
Source: BMC Womens Health. 2026 May 1;26:334. doi: 10.1186/s12905-026-04486-z (PMC13339861; doi:10.1186/s12905-026-04486-z)
Supplement: Supplementary file 1 — Supplementary Material 1. [file 12905_2026_4486_MOESM1_ESM.docx]

**Photovoice Focus Group Protocol**

| **Topic** | **Objectives** | **Participant Tasks & who is leading them** |
| --- | --- | --- |
| Session 0 | Ensure that participants are technologically supported | - Go through a zoom session with them to practice - Show how to use Canvas and Zoom - Provide with demographic survey |
| [Session #1](https://docs.google.com/presentation/d/1SUDRvr00Pu08BfTUj7TePL9yNof8imLrN58Y6CPu1Z8/edit?usp=sharing): [PHOTOVOICE 101](https://docs.google.com/presentation/d/1SUDRvr00Pu08BfTUj7TePL9yNof8imLrN58Y6CPu1Z8/edit?usp=sharing) | 1. Synchronous group introduction, setting the stage, building trust & rapport 2. Developing group norms 3. Provide participants with prompt examples | - Introductions: sessions are recorded so please put in acronyms, preferred acronym/name for meetings, preferred pronouns, and how would you or a health professional you work with define your chronic pain (JP) - Participate in building group norms (RR) - Ask them what Photovoice is? (RR) - Follow up with a brief overview of Photovoice (RR) - Why are they interested in participating or why are they here? Your experience vs (JP) - Decide on prompts (RR & JP) - Remind them to take as many pictures based on prompt (RR) - Picture taking and quality of pictures, including taking consent for people whose picture you are taking (JP) |
| Session #2: The ORID Method | 1. Discuss who took what photos & why (ORID) | - Facilitation of the following ORID questions (RR):   - How many pictures did you take?   - How long did it take you to finish taking pictures?   - What was the most challenging part of taking these pictures?   - How did you feel when you took that picture? - JP call people in one to one via chat - Request to Select 4-6 photos that most reflected the issue they had identified when experiencing chronic pain (RR) |
| [Session #](https://drive.google.com/open?id=1jgR8DKGA42HJpbpIeJS4XI1uvOSprzQV)3: [THE SHOWED METHOD](https://drive.google.com/open?id=1jgR8DKGA42HJpbpIeJS4XI1uvOSprzQV) | 1. Asychronous “The SHOWeD Method”  (Video #2) | - Discussion around SHOWED (RR) using the following questions:   - 1. What do you see here?   - 2.What’s really happening here?   - 3.How does this related to your lives?”   - 4. What can we Do about it? What needs to change?   - Who should be involved in changing these issues? - JP make notes on possible emerging themes at same time. Remind participants to say the number of their picture so we can make the transcription and picture-matching make sense. - ID Themes & Subthemes w/ facilitators. - Read out aloud the themes and subthemes (JP start this off) |
| [Session #](https://drive.google.com/open?id=12dgJ9Fcpl3pgRZnzO16bu_eEGy0N2jjf)4: REFINING IMAGE & TEXT SELECTION | 1. Theme Discussion | - Confirm whether the themes or sub-themes exist in their selections. Ask if there are any other themes to add, eliminate or combine/collapse (JP) - Place pictures and narratives in a PowerPoint and present back to group live (RR) - Participants confirm narrative (RR) - Participants put their photos into themes: JP set up MIRO whiteboard with themes & thumbnails of photos that participants can move around |
| [Session #](https://drive.google.com/open?id=1WB5JQnCpc5nCAXmlKaFv8TBtzxr4dAdj)5: Theme review | 1. Presenting final compilation for group review | - Continue the process as in Session 4 (RR) - Participants look at the compilation by theme and sub theme and edit as a group (JP) |
| [Session #6](https://drive.google.com/open?id=1BOVPzzspXddyh6eTHVz_EZY8lhhoxR-k) -[FINAL SESSION](https://drive.google.com/open?id=1BOVPzzspXddyh6eTHVz_EZY8lhhoxR-k) | 1. Reflection process | - Participants plan about how to share works (JP), and what are the next steps for the project (RR) - The group is to reflect on their experiences of photovoice against the backdrop of their reflective entries. Here are some probe questions (JP):   - 1. What did you like most about participating in this Photovoice project?   - 2.What did you like least about participating in this Photovoice project?   - 3. What types of skills did you develop by learning about Photovoice?   - 4. Do you feel like your perspectives were valued by the rest of the group?   - 5.Do you feel like you have gained confidence and/or comfort by participating in social change activities? - How would you change this project if you were to participate again? - Thank them for their participation and we should reflect as facilitators to share what we learned through their narratives. - Ask them to fill facilitator evaluation survey during the final session |

**Demographic Survey**

| 1. What is your current residence? |  |
| --- | --- |
| 2. How old are you? | _ _  [ineligible if under 18 years old] |
| 3. How do you identify your race, using the following categories?  [Select all that apply.] | White  Black/African American  American Indian/Alaska Native  Asian  Native Hawaiian/Pacific Islander  Prefer not to answer  Other, specify |
| 4. How do you identify your ethnicity? | Hispanic/Latino  Not Hispanic/Latino  Prefer not to answer  Other, specify |
| 5. Where were you born? | United States  Canada  China  Dominican Republic  El Salvador  Guatemala  Honduras  India  Mexico  Philippines  Russia  South Korea  Taiwan  Vietnam  United Kingdom  Other, specify _________ |
| 6. With which religion(s) do you identify? | Christian  Jewish  Muslim  Hindu  Sikh  Jain  Buddhist  Agnostic  Atheist  No religion  Other, specify____________ |
| 7. How many years have you lived in the United States? | [Numerical response only] |
| 8. What are the primary language(s) you speak at home? | English  Spanish  Arabic  Cantonese  French  German  Hindi  Korean  Mandarin  Portuguese  Russian  Tagalog  Vietnamese  Yiddish  Other, specify _________ |
| 9. What is the highest level of education that you have completed? | Less than a high school degree  High school degree  Some college  College degree  Graduate degree |
| 10. What is the sex you were assigned at birth? | Male  Female |
| 11. What is your current gender identity? | Male  Female  Transgender Male  Transgender Female  Gender-nonbinary/Genderqueer  Prefer not to say  Other, __________ |
| 12. What is your sexual orientation? | Heterosexual/straight  Homosexual/gay  Bisexual  Pansexual  Asexual  Queer  Prefer not to say  Other, __________ |
| 13. What is your partnership status? | Single  Married  Civil union/domestic partnership/common law marriage  Living with partner  Partnered but not living together  Widowed  Divorced  Separated |
| 14. If you are partnered, what is your partner’s gender? | Man  Woman  Transgender Man (female to male FTM)  Transgender Woman (male to female or MTF)  Genderqueer  Gender nonconforming  Prefer not to say  Other, specify ___________ |
| 15. How many people are living in your household? | [Numerical response only] |
| 16. What is your employment status? | Employed, part-time  Employed, full-time  Student  Disabled  Retired  Unemployed |
| 17. What is your annual income? | Less than $10,000  Between $10,000 - $20,000  Between $20,000 - $30,000  Between $30,000 - $40,000  Between $40,000 – $50,000  Between $50,000 –$75,000  Between $75,000 –$100,000  Between $100,000 –$150,000  Between $150,000 –$250,000  Over $250,000 |
